# Treatment and unmet needs in steroid-refractory acute graft-versus-host disease

**DOI:** 10.1038/s41375-020-0804-2

**Published:** 2020-04-03

**Authors:** Florent Malard, Xiao-Jun Huang, Joycelyn P. Y. Sim

**Affiliations:** 1 Sorbonne Université, INSERM, Centre de Recherche Saint-Antoine (CRSA), UMRS_938, AP-HP Hôpital Saint-Antoine, F-75012 Paris, France; 2 Peking University People’s Hospital, Peking University Institute of Hematology, Beijing Key Laboratory of Hematopoietic Stem Cell Transplantation, National Clinical Research Center for Hematologic Disease, Beijing, China; 3 Department of Medicine, The University of Hong Kong, Queen Mary Hospital, Hong Kong, China

**Keywords:** Haematological diseases, Drug development

## Abstract

Acute graft-versus-host disease (aGVHD) is a common complication of allogeneic hematopoietic stem cell transplantation (alloHCT) and is a major cause of morbidity and mortality. Systemic steroid therapy is the first-line treatment for aGVHD, although about half of patients will become refractory to treatment. As the number of patients undergoing alloHCT increases, developing safe and effective treatments for aGVHD will become increasingly important, especially for those whose disease becomes refractory to systemic steroid therapy. This paper reviews current treatment options for patients with steroid-refractory aGVHD and discusses data from recently published clinical studies to outline emerging therapeutic strategies.

## Introduction

Acute graft-versus-host disease (aGVHD) is a common complication of allogeneic hematopoietic stem cell transplantation (alloHCT), occurring in ~30–50% of patients, with 14–36% developing severe aGVHD [1]. Onset of aGVHD classically occurs within 100 days of transplant; however, late-onset aGVHD may present after 100 days [2]. aGVHD is a major cause of morbidity, and opportunistic infections are prevalent [3, 4]. Mortality is also high, with only 25–30% of patients with grade III aGVHD and 1–2% of patients with grade IV aGVHD surviving long term (>2 years) [5].

Systemic steroid therapy is the standard first-line treatment for aGVHD [6–8]. However, in ~35–50% of patients, aGVHD becomes refractory to systemic steroid therapy [9, 10]. Mortality is high and quality of life is poor in these patients, and only one treatment is currently approved for steroid-refractory aGVHD (SR-aGVHD) [7, 11]. This review focuses on the unmet need and current and emerging therapies for patients with SR-aGVHD.

### Overview of aGVHD

aGVHD occurs primarily in the skin, gastrointestinal tract, and liver and can occur in alloHCT recipients despite prophylaxis [3]. Patients usually present with a maculopapular rash, nausea, vomiting, profuse watery diarrhea and abdominal cramping, and hyperbilirubinemia with jaundice [2, 11]. The incidence and severity of aGVHD depend on a variety of risk factors, but it occurs more frequently with increased severity after alloHCT from HLA-nonidentical or unrelated donors than from HLA-matched sibling donors [8, 12]. Non-HLA risk factors associated with aGVHD include older patient and/or donor age, use of female donor for male recipient, use of peripheral blood as stem cell source, nature of GVHD prophylaxis, and recipient seropositivity for cytomegalovirus [11, 13].

### Diagnosis and staging

aGVHD is diagnosed clinically after laboratory analysis, imaging, and/or endoscopic examination to exclude potential differential diagnoses. Biopsy may help confirm the diagnosis but lacks sensitivity and specificity. Following diagnosis, aGVHD severity is graded from mild (grade I) to very severe (grade IV) [5]. The modified Glucksberg staging system is considered the standard in the field [5]; however, clinical staging of aGVHD differs between hospitals, making comparisons between clinical studies difficult and possibly contributing to the reason that promising treatments often fail to show benefit in randomized, multicenter clinical trials [14]. However, differences in staging seen across studies occur because of the interpretation and application of the staging system, not the staging system itself. For example, clinicians may have alternative explanations for skin rash, diarrhea, or raised bilirubin level; subjective differences exist in area estimation of skin rash by the rule of nines; and various means are used to stage transaminitis for liver-only aGVHD in the absence of raised bilirubin level. In addition, biopsy of the affected organ may not be immediately feasible due to center and patient factors. Hence, even though guidelines for aGVHD staging based on symptom severity are well established, quantification of the severity of symptoms has not been standardized across centers.

To improve reproducibility, guidelines have been developed through international expert consensus to standardize clinical trial data collection of aGVHD symptoms for use in a large international GVHD research consortium (Mount Sinai Acute GVHD International Consortium [MAGIC]). These new guidelines provide accurate information on the absolute quantification of symptoms that should be included in clinical staging of aGVHD to facilitate future retrospective studies [14] and could be used as a tool to standardize data collection for research. Furthermore, the Transplant Complications Working Party of the European Society for Blood and Marrow Transplantation and the US National Institutes of Health developed an electronic tool, the eGVHD App, based on MAGIC criteria, to assist healthcare professionals in the assessment of GVHD in clinical practice [15]. Although validation of these guidelines is still needed, increased standardization of aGVHD symptom quantification may lead to enhanced clinical study reproducibility. In addition, consensus is growing for adoption of the day 28 response assessment as a short-term primary endpoint in aGVHD studies [16].

### Pathophysiology

**Fig. 1:**
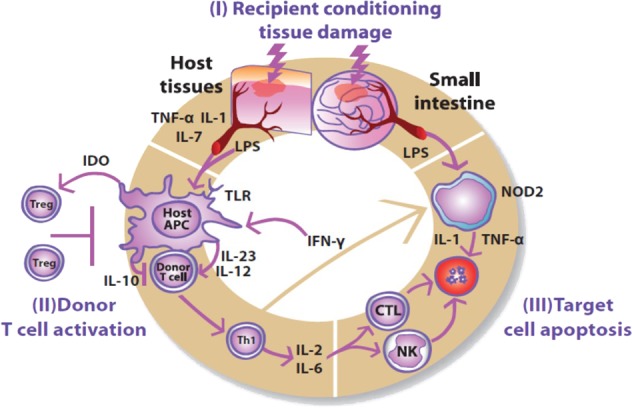
Pathophysiology of acute graft-versus-host disease. During phase I, conditioning chemotherapy or radiation damages tissues and causes release of inflammatory cytokines; during phase II, donor T cells are activated; and in phase III, T cells migrate to target tissues and cause apoptosis. APC antigen-presenting cell, CTL cytotoxic T lymphocyte, IDO indoleamine 2,3-dioxygenase, IFN interferon, IL interleukin, LPS lipopolysaccharide, NK natural killer cell, NOD2 nucleotide-binding oligomerization domain–containing protein 2, TNF tumor necrosis factor, Treg regulatory T cell. From Harris et al. Br J Haematol. 2013;160:288–302. © 2012 Blackwell Publishing Ltd.

The pathophysiology of aGVHD is typically described in 3 phases (Fig. 1). During phase I, conditioning treatment damages patient tissues and causes release of inflammatory cytokines, including tumor necrosis factor (TNF)-α, interleukin (IL)-6, and others that lead to activation of host antigen-presenting cells [13, 17]. During phase II, antigen-presenting cells activate mature donor cells through IL-12 and IL-23 to produce T helper cell type 1 (Th1) cytokines, such as IL-2, IL-6, and interferon γ [13, 17]. T cells migrate to target tissues and cause tissue destruction during phase III. Th1 cells then promote proliferation and differentiation of cytotoxic T lymphocytes and stimulate natural killer cells, which induce apoptosis [13, 17]. The process is also regulated by other parts of the immune system, such as regulatory T cells (Tregs) and invariant natural killer T cells [18–20].

A deeper understanding of the pathophysiology of aGVHD has already resulted in the development of targeted therapies, and new findings may lead to the development of even more. For instance, some therapies target T-cell activation by using antibodies against the IL-2 receptor (IL-2R) [21], whereas others target the third phase of aGVHD, using antibodies against various markers on T lymphocytes [22]. A number of studies also demonstrated that gut microbiota dysbiosis is associated with SR-aGVHD [23–27]. These studies validated that gut microbiota may modulate systemic alloimmune responses and showed that after alloHCT, the abnormal gut microbiota damages gastrointestinal mucosa and ultimately collapses the diversity of intestinal microbiota. A promising new therapy, fecal microbiota transplant (FMT), may restore the microbiota system [23–27]. Another study found that continued tissue damage in patients with intestinal aGVHD led to increased enterocyte proliferation and significant telomere loss [28]. Telomere loss has been associated with replicative exhaustion and tissue failure and might contribute to treatment failure in intestinal aGVHD, providing a basis for stem cell–protective therapies. An additional study suggested that endothelial cell damage also may play a role in the pathogenesis of steroid resistance in aGVHD [29]. Pretransplant levels of angiopoietin-2, a mediator of endothelial vulnerability, correlated with the risk of developing SR-GVHD in patients with ongoing GVHD. The measurement of pretransplant markers of endothelial vulnerability could help determine the transplantation strategy in patients with high risk for developing GVHD.

### Steroid-refractory aGVHD

The recommended first-line treatment for aGVHD is systemic steroid therapy [7, 30]; however, ~35–50% of patients become refractory to steroid therapy [10, 31]. SR-aGVHD can be defined as a clear progression after 3–5 days of treatment or no response after 5–7 days of treatment; [6, 7] however, the exact definition can vary by center. SR-aGVHD is associated with a high mortality risk [8, 10, 32, 33]. The weighted average 6-month survival estimate across 25 studies was 49%; [8] another study reported an estimated 2-year survival rate of 17% [10]. Patients with SR-aGVHD also had higher nonrelapse mortality at 18 months (63% vs 34%) and 2 years (65% vs 35%) than patients who responded to steroid treatment [10]. Likewise, infection-related mortality was high in patients with SR-aGVHD. In a retrospective study of 127 adults, the 1-year incidence of bacterial, viral, and fungal infections was 74%, 65%, and 14%, respectively. Infection-related mortality and overall survival (OS) at 4 years were 46% and 15%, respectively. Bacterial infection was the most common infection leading to death [32].

### Treatments for SR-aGVHD

There is no accepted standard-of-care treatment for SR-aGVHD [11, 34]. This is due to most studies in SR-aGVHD being retrospective, single-arm, phase 2 studies [8, 11, 35], which cannot be easily compared with current patient populations due to significant changes in not only supportive care but also prophylaxis of GVHD. In addition, there are a lack of standardized endpoints, small numbers of enrolled patients, and decreased survival rates, making it difficult to compare data across studies [6, 8]. Consequently, treatment choices are based on physicians’ experiences, taking into consideration GVHD prophylaxis as well as the risks of potential toxicity and exacerbation of preexisting comorbidities [11, 34].

The joint working group established by the British Committee for Standards in Hematology and the British Society for Bone Marrow Transplantation outlined the following options for therapy in SR-aGVHD based on critical review of available literature: extracorporeal photopheresis (ECP), anti–TNF-α antibodies, mechanistic target of rapamycin kinase inhibitors, mycophenolate mofetil, methotrexate, or anti–IL-2R antibodies [6]. They recommended that patients who experience failure of one second-line therapy try another before moving on to third-line options.

The American Society for Blood and Marrow Transplantation (ASBMT) reviewed various therapies (e.g., methotrexate, mycophenolate mofetil, ECP, anti–IL-2R antibodies, alemtuzumab, antithymocyte globulin [ATG], etanercept, and infliximab) and also developed recommendations for treatment of SR-aGVHD [8]. The ASBMT concluded that the choice of a second-line therapy should be based on the effects of any previous treatment and consideration of potential toxicity with treatments. In addition, they supported participation in well-designed clinical studies. The following sections review current treatments for SR-aGVHD (Table 1).

**Table 1:** Treatments for SR-aGVHD.

Treatment	Mechanism of action	Response rate (%)	Toxicity (%)
ECP [38, 98]	Apheresis-based immunomodulator that has immunosuppressive effects against T cells	70–77	Infection (53) Anemia (25) Diarrhea (20) Nausea (18)
ATG [39, 41–45, 99]	Immunomodulatory activity of T-cell depletion; induction of apoptosis in B-cell lineages; induction of Tregs and natural killer T cells	41–57	Fever and infection (≥80)
Daclizumab [46, 48, 51, 52]	Humanized monoclonal antibody against IL-2Rα; inhibits activated T cells	17–82	Infection (up to 95)
Basiliximab [21, 55–59]	Chimeric monoclonal antibody against IL-2Rα; inhibits activated T cells	71–92	Any infections (0–65); CMV reactivation (0–29)
Inolimomab [60, 61]	Murine monoclonal antibody against IL-2Rα; inhibits activated T cells	58–63	No drug-related toxicity; infectious events (93)
Infliximab [62–64]	Anti–TNF-α monoclonal antibody	46–90	Infection (90)
Etanercept [32, 33, 68–70]	Anti–TNF-α monoclonal antibody	28–55	Infection (67–87)
Etanercept plus			
Basiliximab [22]	Etanercept: anti–TNF-α monoclonal antibody Basiliximab: anti–IL-2Rα antibody inhibiting T-cell activation	91	Cytopenias (49; grade 3/4, 32) Hemorrhagic cystitis (28) Invasive pulmonary fungal infection (36 [cumulative incidence at 12 mo]) CMV reactivation (57) EBV reactivation (6)
MSC [72, 73, 100]	Immunomodulatory activity of IL-10	50–83	Acute transient nausea/vomiting and blurred vision during infusion (4)
Methotrexate [74, 75]	Inhibits dihydrofolate reductase and production of thymidylate and purines; suppresses T-cell response, proliferation, and expression of adhesion molecules	58–70	Grade 3/4 hematologic toxicity (42) Grade 3 elevation of transaminases (4)

*ATG* antithymocyte globulin, *CMV* cytomegalovirus, *EBV* Epstein-Barr virus, *ECP* extracorporeal photopheresis, *IL* interleukin, *IL-2Rα* α chain of IL-2 receptor, *MSC* mesenchymal stem cell, *TNF-α* tumor necrosis factor α, *Tregs* regulatory T cells.

### ECP

ECP is widely used for treating SR-aGVHD and consists of exposing peripheral blood mononuclear cells to photoactivated 8-methoxypsoralen, followed by reinfusion of treated cells [36]. ECP is generally considered a safe and effective method for treating SR-GVHD and was associated with superior survival in patients with grade II SR-aGVHD (hazard ratio [HR], 4.6; *P* = 0.016) compared with anti-cytokine therapy; grade > II SR-aGVHD at onset of salvage therapy was associated with inferior survival (HR, 9.4; *P* < 0.001) [37].

A meta-analysis of prospective clinical trials evaluating ECP in patients with SR-aGVHD reported an overall response rate (ORR) and complete response (CR) rate of 71% each [38]. The pooled response rates for skin, liver, and gut SR-aGVHD were 86%, 60%, and 68%, respectively. However, evidence was insufficient to assess benefit due to limited enrollment (*N* = 121). The authors concluded that ECP could be an effective therapy for skin SR-aGVHD. Results were also promising for liver and gut SR-aGVHD, but large double-blind clinical trials are needed to verify these findings.

### ATG

ATG is frequently used for SR-aGVHD, in part due to the Minnesota study [39] and a randomized study comparing prednisone with ATG + prednisone [40]. However, several studies have suggested that it may not be an effective option in SR-aGVHD. For instance, a study assessing long-term survival of patients with grade III/IV aGVHD treated after 2000 found that 41% of steroid-refractory patients (14/34) treated with ATG had a response at 4 weeks, but this decreased to 21% at 12 weeks; 4 patients (12%) were alive at the time of the analysis (range, 3.6–12.8 years posttransplant) [41]. Similar findings were reported in a 20-year (1992–2012) retrospective study [42]. An overall response was observed in 43% of patients; however, OS was poor (5.5 months), with high mortality rates due to infection. These studies suggest that although ATG leads to an initial response, especially in patients with skin-only GVHD, that does not translate to prolonged OS.

More recent studies have reported better outcomes with ATG, which may reflect improvements in contemporary prophylaxis and care regimens. In a prospective, randomized study evaluating ATG vs ABX-CBL, an antibody targeting CD147, the ORR with ATG was 57%, and survival at 18 months was 45% [43]. A phase 3, randomized trial comparing inolimomab (an anti–IL-2R antibody) with ATG found a 1-year survival rate of 40% in the ATG arm [35, 44]. In addition, a recent, small, retrospective study (*N* = 11) reported positive outcomes with low-dose ATG with gradual dose escalation based on response (response-guided ATG therapy). The overall improvement at day 28 was 55%, with rates of OS and transplant-related mortality at 1 year of 55% and 45%, respectively, suggesting that this approach may be necessary when treating patients with ATG [45]. The use of ATG for SR-aGVHD in patients who received ATG as part of their conditioning therapy should be studied to determine the effects of therapy.

### Antibodies against IL-2R: daclizumab

Retrospective studies showed that daclizumab, a humanized monoclonal antibody against the α chain of IL-2R, had activity in SR-aGVHD (ORR, 51–67%), especially in patients with skin or mild gut aGVHD, but was associated with an increase in infectious complications and poor long-term outcomes [46, 47]. In a phase 2 study of daclizumab in SR-aGVHD (*N* = 62), 69% of patients had a CR on day 30, and 21% had a partial response (PR); the 4-year event-free survival was 55% [48]. Lower event-free survival was seen in patients with grade ≥III SR-aGVHD, in those with ≥2 involved organs at baseline, and those who were >18 years old [48]. Similar results were reported in other studies, including in pediatric patients; [46, 49, 50] however, one study reported poor efficacy (17%), possibly due to GVHD severity [51]. Despite encouraging responses, rates of infectious complications were high (up to 95% in one study), and long-term survival was poor [48–52].

Findings from a small, retrospective study suggest that daclizumab may lead to a better response if administered in combination with other agents and with aggressive infection prophylaxis [50]. In this study, all patients treated with daclizumab alone (*n* = 6) or in combination with ATG or infliximab (*n* = 6) achieved a CR and had a Kaplan–Meier probability of survival at 100 days of 100% [50]. Other studies also found the combination therapy to be effective (ORR, 47–86%) [53, 54], but survival was not always improved. No randomized studies with daclizumab in GVHD are currently ongoing.

### Antibodies against IL-2R: basiliximab

Basiliximab, a chimeric monoclonal antibody against the α chain of IL-2R, was first reported to be effective and safe for the treatment of SR-aGVHD in a study of 17 patients with skin, liver, and/or intestinal SR-aGVHD [55]. Treatment with basiliximab led to an ORR of 71%, with 53% CR (median follow-up, 123 days). No bacterial or fungal infections were observed; 5 of 17 patients had a cytomegalovirus reactivation. A prospective phase 2 study (*N* = 23) also reported a high ORR (83%); however, the rate of CR was lower (18%) [56]. Infections occurred in 65% of patients in this study; 48% of patients were alive after a mean follow-up of 2 years. Several subsequent studies also showed high ORRs (82–92%), including in pediatric patients; however, ~50–70% of patients experienced GVHD recurrence, and rates of infectious complications were high [21, 57–59]. Kaplan–Meier-estimated probabilities were 48% for 3-year event-free survival and 20% for 5-year OS [21, 57].

### Antibodies against IL-2R: inolimomab

Retrospective studies suggested that inolimomab, a monoclonal antibody that inhibits the α chain of IL-2R, might be an effective therapy for patients with SR-aGVHD, particularly those without gastrointestinal involvement [60, 61]. The ORR in these studies ranged from 58 to 63%, and OS was 30% at 1 year and 26% at 3 years. No drug-associated toxicity was reported, although 93% of patients had ≥1 infectious event in one study [61], and 14% of patients died due to infections in another [60]. Inolimomab was recently evaluated in a phase 3, randomized, open-label, multicenter trial that compared inolimomab (*n* = 49) with ATG (*n* = 51) in adult patients with SR-aGVHD [35]. The primary endpoint of therapy success, defined as OS at 1 year without changing baseline therapy, was not met and was reached by 14 patients treated with inolimomab (29%) and 11 patients (22%) treated with ATG (adjusted HR, 0.722; *P* = 0.188); 53% and 60% of patients died in the inolimomab and ATG groups, respectively. A long-term follow-up analysis of this study (median follow-up, 58.4 months) showed a clinical benefit associated with inolimomab compared with ATG (31% vs 20% survival; adjusted HR, 0.572 [95% CI, 0.346–0.947]; two-sided *P* = 0.030) [44]. The number of deaths related to infection was two times lower in patients treated with inolimomab than with ATG (12% vs 24%). Although findings are promising, further studies are needed to determine the benefits of inolimomab compared with other therapies.

### Anti–TNF-α antibodies: infliximab

Infliximab has shown mixed results for the treatment of SR-aGVHD [62, 63]. In a retrospective study (*N* = 68; 51 patients [75%] with grade III/IV), 41 patients (60%) showed response to infliximab therapy at day 7, and 31 patients (46%) showed response at day 28 [62]. Twenty-four patients (35%) achieved the composite endpoint of 6 months’ freedom from treatment failure, and 34% of patients were alive at 24 months. However, infections occurred in 61 patients (90%) and infections led to death in 17 patients (33%). The main cause of treatment failure within 6 months was death (31 patients). In a small study, nine of ten patients responded to treatment; however, four patients died of sepsis [64]. A recent study showed that infliximab therapy in SR-aGVHD was associated with a modest, poorly sustained response along with an increased risk of severe infection [63]; other studies showed similar results [65].

### Anti–TNF-α antibodies: etanercept

Early studies evaluating etanercept, a second anti–TNF-α antibody, in combination with other agents for the treatment of SR-aGVHD reported high response rates of 67 [66] and 81% [67]. In the first retrospective study to evaluate etanercept monotherapy in SR-aGVHD, etanercept led to a clinical response rate of 46% (6/13) in patients with SR-aGVHD, with the highest response rates seen in patients with gastrointestinal involvement (64%; 7/11). A survival benefit was also observed; 69% of patients were alive at a median follow-up of 10.6 months. Common infections were cytomegalovirus reactivation (48%), bacterial infections (14%), and fungal infections (19%) [68]. Subsequent studies found response rates to be similar (50–55%) [32, 69, 70], although a retrospective study with a median follow-up of 74 months reported a clinical response rate of 28% [33]. Despite inducing response, etanercept showed little to no improvement in OS in these studies (0–28%) [32, 33, 69, 70]. However, these were all small (<30 patients treated with etanercept in each), retrospective studies, which may have affected results.

Although the rate of infectious complications was high in these studies (up to 87%), etanercept is usually associated with lower rates of infection compared with infliximab [32, 68–70]. This may be due to the different mechanisms of action of the two agents, in particular the cytolytic activity observed with infliximab but not etanercept; however, no direct comparison between etanercept and infliximab has been performed [68].

### Combination of anti–TNF-α and anti–IL-2R antibodies

Basiliximab in combination with etanercept was assessed in a prospective, multicenter clinical trial of 65 patients with severe (grades III–IV) SR-aGVHD [22]. The ORR at day 28 was 91%; 75% of patients had a CR. The 2-year OS rate was 55% [22], suggesting that combination therapy may further improve outcomes in patients with SR-aGVHD. However, this clinical benefit may be limited to basiliximab plus etanercept, given that a retrospective study of combination therapy with inolimomab plus etanercept only showed an ORR of 48% at day 28 and a 2-year OS of 10% [71]. Similarly, a study of basiliximab plus infliximab led to lower response rates (ORR, 76%; CR, 43%) and worse survival (24% at 1 year) than basiliximab alone [65].

### Third-line options

Treatments with fewer data available are considered to be third-line treatment options and include alemtuzumab (anti–CD52 receptor antibody), pentostatin, mesenchymal stem cells (MSC), and methotrexate [6]. However, studies have suggested that MSC and methotrexate are promising therapies. Two recent studies of MSC reported positive outcomes [72, 73]. One retrospective study of 46 patients with grade III/IV SR-aGVHD reported a 50% response rate (3 patients had a CR, 14 had a PR, and 6 had a transient PR), and the estimated probability of survival at 2 years was 17% [73]. The other, a prospective study of 69 patients (51 children, 18 adults), reported an ORR of 83% at day 28 (22 patients had a CR, 35 patients had a PR), and the 6-month OS probability was 71% [72]. However, the use of MSC may be difficult in some countries because of regulatory issues.

Similarly, in a retrospective study of low-dose methotrexate for SR-aGVHD, 7 of 12 patients (58%) responded (5 had CR) [74]. Eight patients had grade III/IV SR-aGVHD, with five patients responding to low-dose methotrexate. Seven patients died of disease progression (nonresponders, four patients; responders, three patients). In a pooled data analysis, the observed overall response was 70% (79/113 patients), with a CR in 59% of patients and a PR in 11% [75]. Pooled data suggested the potential use of methotrexate for SR-aGVHD; however, randomized controlled studies are needed. In both studies, methotrexate toxicity was low [74, 75].

The use of other therapies has been reported; however, evidence is insufficient to support recommending their use in the management of SR-aGVHD [6]. These agents include rituximab (anti-CD20), visilizumab (anti-CD3), ABX-CBL (anti-CD147), thalidomide (immunomodulatory drug), azathioprine (purine analog), intra-arterial methylprednisolone, and Tregs.

## Novel therapies evaluated in recent clinical studies

### Ruxolitinib

Ruxolitinib is a Janus kinase (JAK) 1/JAK2 inhibitor that became the first JAK inhibitor approved for the treatment of myelofibrosis [76]. JAKs are intracellular signaling molecules and are important effectors of all three phases of the pathogenesis of aGVHD [77–80]. Ruxolitinib’s inhibition of JAK1/JAK2 influences a wide range of immune system components, both adaptive (dendritic cells and CD4^+^ T cells) and innate (natural killer cells and neutrophils) [81]. In preclinical studies, ruxolitinib reduced the severity of GVHD and prolonged survival in animal models of GVHD while preserving the graft-versus-leukemic effect through inhibition of the production of proinflammatory cytokines, suppression of T-cell expansion, and promotion of Treg proliferation (Table 2) [77, 79].

**Table 2:** Novel therapies for SR-aGVHD evaluated in recent clinical studies.

Treatment	Mechanism of action	Response rate (%)	Toxicity (%)	Ongoing clinical studies
Ruxolitinib [77–80, 82, 84]	JAK1/JAK2 inhibitor	45–82	Anemia (60) Hypokalemia (48) Decreased platelet count (44) Peripheral edema (44) Decreased neutrophil count (37) Cytomegalovirus (13) Viremia (6) Chorioretinitis (1)	NCT02953678 NCT02913261 NCT02396628
FMT [23–27]	Reestablishes the microbiota system through infusing a fecal suspension from a healthy donor into a patient’s gastrointestinal tract	100	Abdominal pain and diarrhea (100)	NCT03148743 NCT03812705 NCT03359980 NCT03214289
AAT [89]	Downmodulates inflammation and increases ratio of Treg to Teff	65	Infections (33) Bacteremia (13) CMV reactivation (5)	NCT03172455
Anti-CD3/CD7 immunotoxin [90]	Induces depletion of T and NK cells; suppresses T-cell receptor activation	60	Worsening of hypoalbuminemia (10), microangiopathy (10), and thrombocytopenia (45) Bacteremia (25) CMV reactivation (15) EBV reactivation (15)	NCT04128319
Vedolizumab [91–93, 95]	Monoclonal antibody to the integrin α4β7 present on circulating lymphocytes, which inhibits their relocation to the gastrointestinal tract	40–100	In ≥3 patients (300 mg; 600 mg): [95] Anemia (50; 33) Nausea (38; 0) Peripheral edema (38; 33) Hypokalemia (38; 56) Hyperglycemia (38; 22) Hypoalbuminemia (25; 33) Dizziness (13; 33) Hypomagnesemia (13; 33) Neutropenia (13; 33) Fatigue (0; 33) Serious infections (≥2 patients): Sepsis (25; 11)	NA

*AAT* α_1_-antitrypsin, *CMV* cytomegalovirus, *EBV* Epstein-Barr virus, *FMT* fecal microbiota transplant, *IL* interleukin, *JAK* Janus kinase, *NA* not applicable, *NK* natural killer, *Teff* effector T cell, *TNF-α* tumor necrosis factor α, *Treg* regulatory T cell.

In retrospective clinical studies, ruxolitinib resulted in fair to high response rates, prolonged survival in patients with SR-aGVHD, and demonstrated a favorable safety profile in these patients [78, 80, 82, 83]. A retrospective survey evaluated outcomes of 95 patients with SR-GVHD (54 with aGVHD, 41 with chronic GVHD) who received ruxolitinib as second-line therapy [80]. The ORR in the SR-aGVHD group was 82% (CR, 46%). The estimated 6-month survival and relapse rate were 79% and 7%, respectively [80]. Long-term follow-up at a median of 19 months showed that 41% of patients had an ongoing response and were free of immunosuppression, with a 1-year OS rate of 62% [83].

A retrospective study of 13 pediatric patients who received ruxolitinib as salvage therapy for SR-aGVHD evaluated response rates after 4 weeks of therapy [82]. Of 11 evaluable patients, one achieved a CR, four had PR, and two had no response; treatment failed in four patients [82]. Seven patients were alive at long-term follow-up at a median of 401 days [82].

Three ongoing studies are evaluating ruxolitinib in SR-aGVHD [78]. The first is the Ruxolitinib in Patients With Refractory GVHD After Allogeneic Stem Cell Transplantation 1 (REACH1; NCT02953678) study, which is an open-label, single-cohort, multicenter, phase 2 study to assess the combination of ruxolitinib with steroids for the treatment of SR-aGVHD (grades II–IV); the primary endpoint is ORR at day 28 [78, 84]. A total of 71 patients were enrolled (median age, 58 years); 68% had grade III/IV GVHD at baseline. The study met its primary endpoint, with an ORR of 55% at day 28 and a best overall response at any time of 73% (CR, 56%). Median duration of response with ≥6 months’ follow-up was 345 days in both day 28 responders and patients who had a best overall response at any time during treatment. In addition, most patients achieved a sustained reduction in steroid dose. The most common hematologic treatment-emergent adverse events (AEs) were anemia (65%), thrombocytopenia (62%), and neutropenia (48%). Infections included cytomegalovirus (13%), sepsis (13%), and bacteremia (10%). Fatal treatment-related AEs were sepsis and pulmonary hemorrhage (1 patient each) and were attributed to both ruxolitinib and steroid treatment. On the basis of this study, ruxolitinib recently became the first US Food and Drug Administration-approved treatment for SR-aGVHD in adult and pediatric patients ≥12 years old [76].

Ruxolitinib is also being assessed in the REACH2 study (NCT02913261), an open-label, multicenter, phase 3 crossover study comparing ruxolitinib with best available treatment (BAT) for SR-aGVHD; the study met its primary endpoint of ORR at day 28 [78, 85]. The third study, Ruxolitinib in GVHD (RIG; NCT02396628), is an open-label, multicenter, prospective, randomized, phase 2 study comparing the efficacy of ruxolitinib plus BAT vs BAT in SR-aGVHD [86].

### Fecal microbiota transplant

FMT is a therapy that reestablishes the microbiota system through infusing a fecal suspension from a healthy donor into a patient’s gastrointestinal tract [87, 88]; three case reports of its use in patients with SR-aGVHD have been published (Table 2) [23–25, 27]. A pilot study of four patients (three with gastrointestinal SR-aGVHD; one with steroid-dependent gastrointestinal aGVHD) evaluated the safety and efficacy of FMT [24]. All four patients responded to treatment (three had a CR; one had a PR). All AEs were mild and transient. The authors noted that peripheral effector Treg counts were increased during the response to FMT. In addition, temporal dynamics of microbiota appeared to be associated with the health of patients’ gastrointestinal tracts. The authors presented another case report of a patient with gastrointestinal SR-aGVHD that also demonstrated restoration of bacterial diversity with no safety concerns following administration of FMT capsules [23]; other case reports showed similar results [25, 27]. A pilot study conducted in eight patients with gastrointestinal SR-aGVHD who were treated with FMT (NCT03148743) [26] showed that all patients’ symptoms improved and bacterial diversity was restored. No severe AEs were reported; four patients died, but the deaths were unrelated to FMT. Progression-free survival improved in the patients who received FMT when the authors retrospectively compared findings with those of patients with gastrointestinal SR-aGVHD who did not receive FMT (*P* = 0.003); however, no difference in OS was observed. Six studies are ongoing to evaluate the efficacy and safety of FMT in patients with gastrointestinal SR-aGVHD (NCT03819803, NCT03549676, NCT03492502, NCT03812705, NCT03359980, and NCT03214289).

### α_1_-Antitrypsin

α_1_-Antitrypsin (AAT), a circulating protease inhibitor produced by the liver, is implicated in several aspects of immune regulation: It inactivates serine proteases from neutrophils and macrophages, induces IL-10, and suppresses plasma proinflammatory cytokines (Table 2) [89]. In murine models, AAT reduced the severity of aGVHD, reducing inflammatory cytokines and increasing the ratio of Tregs to effector T cells—this led to a phase 2 clinical trial in patients with SR-aGVHD (NCT01700036). Forty patients received 60 mg/kg AAT intravenously every 4 days for up to 4 weeks. At day 28, ORR and CR rates were 65% and 35%, respectively [89]. There is an ongoing early-access clinical study (NCT03172455), and AAT is also being investigated as prophylaxis for SR-aGVHD (NCT03459040).

### Anti-CD3/CD7 immunotoxin

An immunotoxin combination, consisting of a mixture of anti-CD3 and anti-CD7 antibodies separately conjugated to recombinant ricin A (CD3/CD7-IT) induces in vivo depletion of T cells and natural killer cells and suppresses T-cell receptor activation (Table 2) [90]. In a phase 1/2 trial of CD3/CD7-IT in SR-aGVHD, 20 patients were given 4-h intravenous infusions of 4 mg/m^2^ CD3/CD7-IT; 4 infusions were given, administered at 48-h intervals. At day 28, ORR and CR rates were 60% and 50%, respectively [90]. CD3/CD7-IT was recently given fast-track designation for the treatment of SR-aGVHD by the US Food and Drug Administration, and a phase 3 trial is ongoing (NCT04128319).

### Vedolizumab

Vedolizumab is a monoclonal antibody that recognizes the integrin α4β7 present on circulating lymphocytes and inhibits their relocation to the gastrointestinal tract [91, 92]. Recent case series have reported mixed results for vedolizumab in gastrointestinal SR-aGVHD (Table 2) [91–94].

Fløisand et al. described a case series of six patients with SR-aGVHD with single-site-involvement, in which all patients responded to second-line treatment with vedolizumab; four patients were alive at a median follow-up of 10 months [92]. However, another case series reported only two PRs from five patients with multiple-site-involvement treated with vedolizumab in the third or fourth line [93].

Another retrospective case series also described a high mortality rate following vedolizumab therapy for gastrointestinal SR-aGVHD: Of nine patients with multiple-site- involvement, six died before the 4-week follow-up, and only one patient survived past 2 months. However, patients surviving past the 4-week follow-up did experience some clinical response [91].

The discrepancies in outcomes with vedolizumab treatment for SR-aGVHD may be related to its mechanism of action: Preventing the migration of lymphocytes to the gastrointestinal tract may be effective only at an early stage and not when GVHD is established. A retrospective case series of 29 patients reported significantly better outcomes in patients treated with vedolizumab in the second vs third or higher line (ORR, 13/13 [100%] vs 10/16 [63%]; *P* = 0.012 and CR, 7/13 [54%] vs 1/16 [6%]; *P* = 0.005) [94]. Nevertheless, a recent dose-finding, prospective, phase 2 study of vedolizumab in the second line (NCT02993783) was terminated early for lack of efficacy [95]. The ORR at day 28 was 50% in patients treated at 300 mg (*n* = 8) and 22% in patients treated at 600 mg (*n* = 9); 12% and 0% of patients, respectively, achieved CR. Consequently, vedolizumab development has been abandoned in the setting of SR-aGVHD. Of note, given vedolizumab’s mechanism of action, development now focuses on GVHD prophylaxis (NCT03657160).

### Other therapies for treating SR-aGVHD

Other promising therapies have also been evaluated in the SR-aGVHD setting but have not shown positive results. Brentuximab vedotin, a CD30-directed antibody drug conjugate, showed promising activity in a phase 1 study in patients with SR-aGVHD [96]. The ORR at day 28 was 38% (CR, 15%); seven additional patients achieved CR by day 56. The OS rate was 41% (95% CI, 25–57%) at 6 months and 38% (95% CI, 22–54%) at 12 months. However, all clinical studies of brentuximab vedotin for the treatment of SR-aGVHD have been terminated or withdrawn. It is currently being evaluated in the prophylaxis of GVHD (NCT01700751).

Begelomab, an antibody targeting CD26 on CD4^+^ T lymphocytes, was in phase 2/3 development for SR-aGVHD. In one study of 28 patients, 75% of patients achieved a response at day 28 compared with 41% of matched controls (*P* = 0.004) [97]. The OS at 1 year was 50% vs 31% in the control group (*P* = 0.06). Although these findings led to further assessment in a phase 3, randomized study (NCT02411084), the study was terminated due to a low accrual rate.

## Conclusions

aGVHD is a major cause of morbidity and mortality in patients undergoing alloHCT. As the number of patients undergoing alloHCT increases, it will become imperative to determine safe and effective treatment options for these patients, especially those who become refractory to systemic steroid therapy. Understanding of the pathophysiology of the disease will continue to be important in determining new targets. As new therapies are developed, demonstrating their efficacy and safety—including long-term effects, such as the incidence of relapse, chronic GVHD, and infections—in large, randomized clinical studies will be imperative. Several emerging therapies are currently being evaluated in phase 3 randomized studies, and first results are eagerly awaited.
